# Immunometabolism: The role of gut‐derived microbial metabolites in optimising immune response during checkpoint inhibitor therapy

**DOI:** 10.1002/ctm2.70472

**Published:** 2025-09-05

**Authors:** Agnieszka Beata Malczewski, Jermaine Ig Coward, Natkunam Ketheesan, Severine Navarro

**Affiliations:** ^1^ Icon Cancer Centre, Wesley and South Brisbane Brisbane Queensland Australia; ^2^ Faculty of Medicine University of Queensland Brisbane Queensland Australia; ^3^ Science and Technology, University of New England Armidale New South Wales Australia; ^4^ Infection and Inflammation Program, QIMR Berghofer Medical Research Institute Brisbane Queensland Australia

## Abstract

Checkpoint inhibitor therapy is the most common type of immunotherapy used in the clinical setting, however, there are significant obstacles with treatment resistance and cancer progression. Since its introduction, there have been relatively few advances in the development of prognostic or predictive biomarkers. The field of metabolomics studies small molecules and can provide us with an understanding of the dynamic events evolving during the tumour‐drug‐immune‐system interaction. Several key pathways have emerged as important in understanding resistance mechanisms thereby providing a rationale for targeting immunometabolism in order to enhance the immune response during checkpoint inhibitor therapy. In the first part of this review, we explore the role of gut microbiome‐derived short‐chain fatty acids, which are recognized as important immunoregulatory molecules shaping T‐cell activation, effector and memory function. We then discuss tryptophan catabolism as a key predictor of primary checkpoint inhibitor failure. Lastly, we focus on immunometabolism as an important future target in immunotherapy.

**One sentence summary:**

This review focusses on microbiome‐derived metabolites and their role in immunometabolism and the enhancement of checkpoint inhibitor responses.

## Graphical abstract

Unique metabolic signatures are associated with both response and resistance to cancer immunotherapy. These signatures can be used to predict response to treatment and in the future, metabolites could potentially be used to enhance specific types of immune response associated with favourable immunological outcomes.

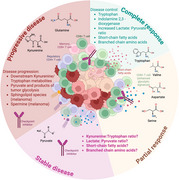

## 1 INTRODUCTION

Checkpoint inhibitor therapy (CIT) is currently a routine treatment for immune‐responsive solid tissue cancers.^1^ Despite over a decade of clinical use and its transformative impact on cancer management, its efficacy remains limited. The majority of patients do not achieve a complete radiological remission, and many will develop secondary CIT resistance.^2^ Given that CIT is likely to remain one of the most common treatments offered to oncology patients, it is important that we optimize the way in which it is used, monitored and adapted for future generations of patients. Clinicians typically employ radiological imaging modalities, including CT and PET, to evaluate patient response. The first imaging‐based assessment is generally performed approximately 3 months after initiation of CIT. Presently, there is a paucity of reliable predictive biomarkers for immunotherapy‐based treatments.^3^, ^4^ Early identification and correction of defects in the patient immune response would provide substantial clinical benefit, particularly through the coordination and enhancement of CD8+ T cell functionality.^5^ Although interest in the gut microbiome as a biomarker has increased, significant challenges remain, such as defining the optimal signature associated with CIT response.^6^, ^7^, ^8^ Increasing evidence suggests that T cell differentiation and function are shaped by metabolites and that metabolic reprogramming of the tumour microenvironment (TME) could be an important adjunct to generating an effective anti‐tumour response.^9^ In this review, we consider the evidence for modulating immunity through metabolites by exploring emerging metabolomics results for patients undergoing CIT. Key areas of focus include microbiome‐derived metabolites such as short chain fatty acids (SCFA), the role of tryptophan catabolism and immunometabolism within the TME itself. We aim to contextualize these results in the modern era of CIT and facilitate potential clinical applications.

## 2 ENHANCING IMMUNITY AND CIT RESPONSES THROUGH MODULATION OF THE MICROBIOTA

Evidence to date suggests that many immunological processes are impacted by the microbiota.^7^, ^10^ Replication of a'favourable microbiome' remains challenging due to the inherent complexity of the microbial ecosystem and its multifactorial influence on host immune function, aspects of which are only beginning to be elucidated. Both gut microbiome diversity and specific microbial composition have been investigated as potential determinants of immune activity.^7^, ^9^ The relationship extends beyond the presence or absence of particular taxa but the overall microbial community architecture, functional gene repertoire and metabolite production.^11^ Novel strategies to modulate the microbiome may include the administration of pre‐ or probiotics, dietary fibre, or SCFA.^7^, ^12^ Emerging evidence suggests these may enhance immune function by promoting beneficial microbial metabolite production capable of supporting CD8+ T cell activity, and potentially improving response to CIT.^7^, ^13^, ^14^, ^15^ Modifying the microbiome through use of probiotics and/or a high fibre diet are both non‐invasive low‐cost interventions, which are already of great interest to patient groups. The proportion of oncology patients taking probiotics has been reported as up to 29%.^7^, ^12^ Spencer et al. (2022) found no statistically significant detrimental effect of probiotic use in patients based on self‐reported data; however, a preclinical melanoma mouse model demonstrated that supplementation with commercially available probiotics impaired anti‐tumour immunity.^7^ Supplementation with *Bifidobacterium longum* or *Lactobacillus rhamnosus* prior to CIT treatment resulted in significantly larger tumours compared to untreated mice (sterile water control), which was associated with a reduction in the frequency of tumour infiltrating IFN γ‐producing CD8 T cells.^7^ Bender et al. (2023) confirmed that *Lactobacillus reuteri* (*Lr*) could facilitate CIT by selectively improving the immunogenicity of the TME.^16^ In this study, the anti‐melanoma effects of four probiotic strains, *Bifidobacterium longum*, *Lactobacillus reuteri*, *Lactobacillus johnsonii* and *Escherichia coli* were evaluated in vivo. Among these, *L. reuteri* exhibited the most potent anti‐tumour activity, which was characterized by the expansion of IFN γ‐producing CD4 T cells and CD8+ Tc1 cells within the TME. This effect was mediated through the release of indole‐3‐aldeyde (I3A), a tryptophan catabolite that activates the aryl hydrocarbon receptor (AhR) on CD8+ T cells, enhancing their anti‐tumour response. Importantly, *L. reuteri* was shown to translocate to and persist within melanoma tumours, where it exerted its effects independently of tumour‐ or bacterium‐specific antigens.^16^ These findings underscore the potential of *L. reuteri* as a therapeutic adjunct and that targeted probiotics or bacterial consortia offer a promizing line of investigation to enhance anti‐tumour immune response.^12^ Although murine models offer valuable insights into microbiome‐immune interactions, their findings may not fully translate to humans due to species‐specific differences in immune system function and microbiota composition. Manipulating the human microbiome through probiotic supplementation requires careful consideration. The efficacy and safety of probiotics in cancer patients remain an area of active research, with some studies suggesting potential benefits, while others highlight concerns regarding their impact on treatment responses and overall health.

## 3 MICROBIOME‐DERIVED METABOLITES: POTENT MODULATORS OF IMMUNE CELL FUNCTION

**FIGURE 1:**
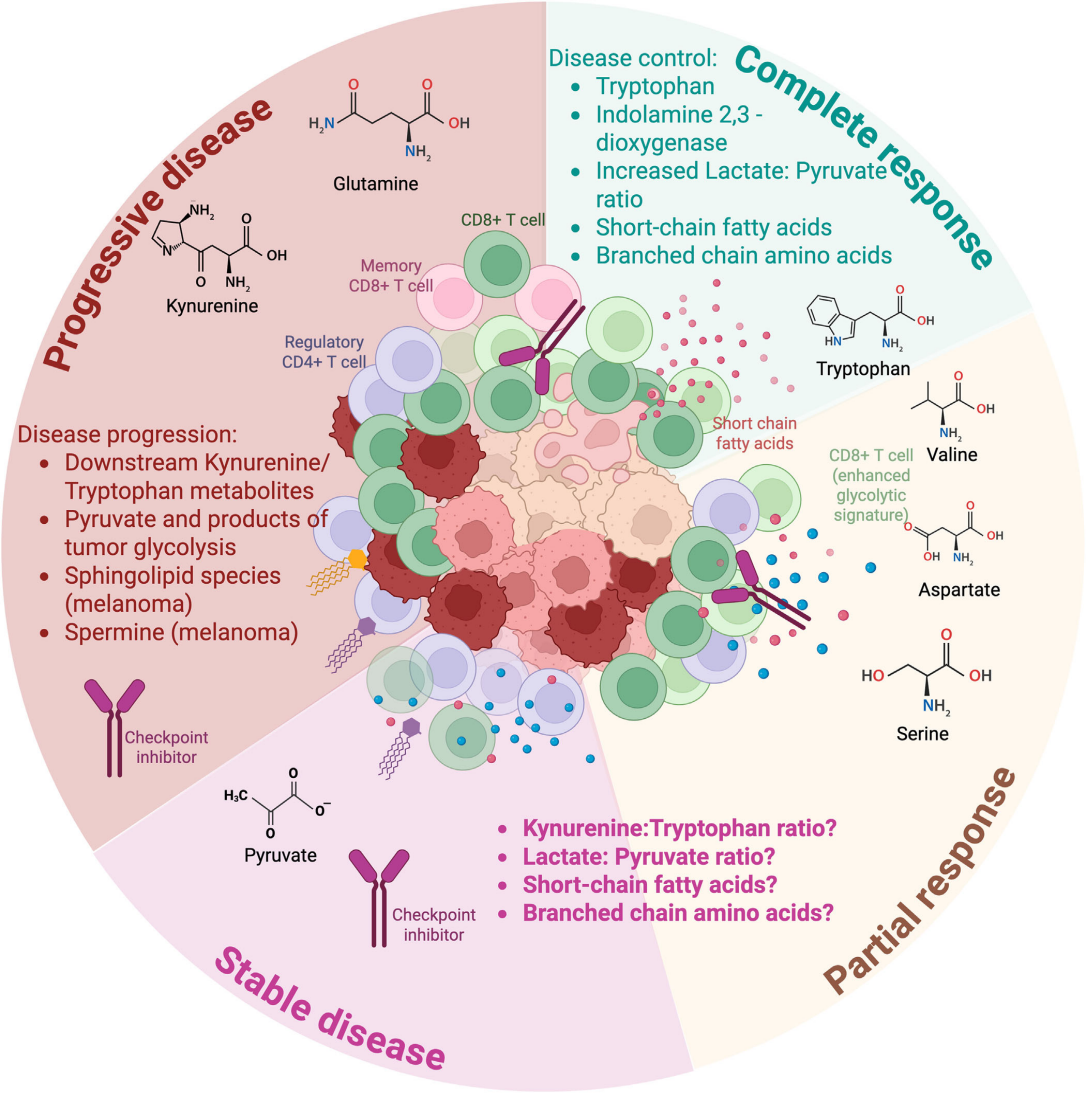
Metabolic signatures are associated with disease response and progression. Unique metabolic signatures are associated with both response and resistance to checkpoint inhibitor therapy (CIT). These signatures can be used to predict response to treatment and in the future, metabolites could potentially be used to enhance specific types of immune response associated with favourable immunological outcomes.

The uncertainty of probiotic supplementation and the challenge of identifying the optimal microbial consortium with proven benefit has led to increasing interest in microbiome‐derived metabolites. These metabolites offer a more precise and controllable approach to modulating immune responses. Metabolomics, the comprehensive analysis of small molecules, allows the detailed interrogation of these pathways in patients receiving CIT, using samples such as blood or faeces. Once considered merely signalling molecules, metabolites are now recognized as potent immunomodulators that shape immune cell differentiation and function, establishing immunometabolism as a promising therapeutic target (Figure 1).^4^, ^17^, ^18^ Key classes of metabolites are currently under investigation including tryptophan, glucose, short‐ and long‐chain fatty acids and amino acids.^19^, ^20^, ^21^, ^22^, ^23^, ^24^ SCFA in particular have been extensively studied for their potential in enhancing CD8+ T cell cytotoxic and memory potential, both of which are central to CIT efficacy.^14^ Dietary fibre is the ideal substrate for SCFA‐producing bacteria and there is evidence that increased fibre intake correlates to increased SCFA production in pre‐clinical model.^12^, ^15^, ^25^ Interestingly, these findings were microbiota dependent and could not be replicated in a germ‐free mouse model. In the clinical part of this study, patients who reported sufficient fibre intake had significantly improved progression free survival (PFS) compared to those who reported insufficient intake. The best performing patient group was characterised by'sufficient fibre intake' and no probiotic intake.^7^

## 4 SCFA ENHANCE CYTOTOXIC T CELL RESPONSES

Short‐chain fatty acids (SCFA) are microbial metabolites produced through the fermentation of dietary fibres and resistant starches in the gut.^15^ These metabolites are essential to maintaining intestinal homeostasis and have profound effects on systemic immune function. SCFA belong to the fatty acid family (FA), which also includes medium and long chain fatty acids and are made up of two to five carbon atoms. Acetate (C2), propionate (C3) and butyrate (C4).^15^ SCFA exert their immunomodulatory effect via multiple mechanisms, notably through the induction of histone deacetylases (HDACs) and activation of G‐protein coupled receptors (GPCRs) such as FFAR2 (GPR43), FFAR3 (GPR41) and GPR109A.^26^

**FIGURE 2:**
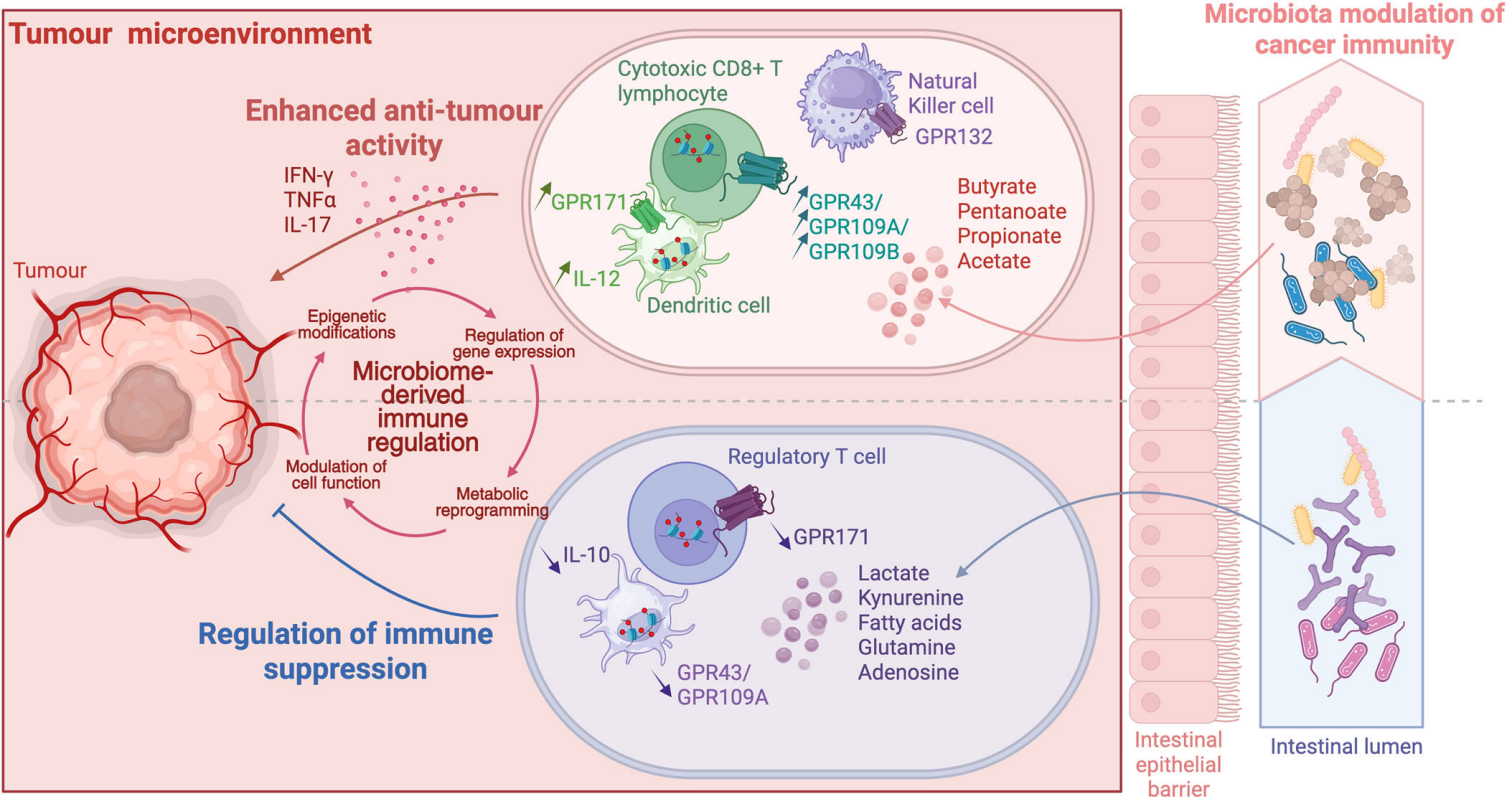
Metabolic reprogramming of immune cells with microbiome‐derived metabolites and anti‐tumour activation. Metabolites produced by commensals can modulate immune cell function via metabolic reprogramming. The production of butyrate, pentanoate, propionate and acetate in particular are important for the activation of anti‐tumour immunity mediated via G‐coupled protein receptors (GPR) on dendritic cells, cytotoxic CD8+ T cells and natural killer cells. In parallel, the modulation of other GRP on regulatory T cells and tolerogenic dendritic cells allow to modulate tumoural immunosuppressive environment.

SCFA influence the differentiation of T cells by modulating epigenetic and metabolic pathways. Inhibition of HDACs in T cells leads to acetylation of histones, resulting in altered gene expression that promotes the development of regulatory T cells (Tregs) or effector T cells, including Th17 and cytotoxic CD8+ T cells (Figure 2).^15^, ^27^, ^28^ In humans, immune response has a spectrum of activity with poor immunosurveillance resulting in cancer escape, whereas immune overactivity and inflammation lead towards autoimmune diseases states.^29^, ^30^ In vitro studies have shown that SCFAs can enhance T cell differentiation and cytokine production. For instance, butyrate supplementation can increase the production of IL‐10 in Tregs or IL‐17 in Th17 cells, thus influencing the balance of pro‐ and anti‐inflammatory signals.^15^, ^27^, ^28^ The impact of SCFAs extends to the enhancement of CD8+ T cells‐mediated anti‐tumour immunity, including the promotion of both effector and memory phenotypes.^13^, ^14^, ^15^ In murine models of melanoma and pancreatic cancer, treatment of cytotoxic T lymphocytes and ROR1‐targeting CAR T cells with butyrate and pentanoate resulted in elevated production of effector molecules such as CD25, IFN‐γ and TNF‐α leading to improved anti‐tumour immunity and reduced tumour volume and weight compared to untreated cells.^14^

Butyrate, in particular, has garnered attention for its potential anti‐neoplastic properties in colorectal cancer.^31^ It has been shown to induce apoptosis in colorectal cancer cells and enhance the efficacy of conventional treatments, including radiotherapy and chemotherapy.^32^ He et al. (2021) reported that butyrate treatment facilitated CD8+ T cell mediated anti‐tumour responses in mice undergoing oxaliplatin chemotherapy.^33^ This study demonstrated that oxaliplatin produced an immunostimulatory effect with increased infiltration of CD8+ T cells into the TME, and that antibiotic‐induced depletion of the gut microbiota with ampicillin, neomycin, vancomycin and metronidazole diminished this effect. Subsequent oral administration of gut microbial metabolites restored CD8+T cell infiltration and IFN‐γ production, underscoring the critical role of gut microbiota‐derived metabolites in modulating chemotherapy efficacy.^33^ Upon activation, effector T cells undergo metabolic reprogramming characterised by an increase in glycolytic activity.^34^ Acetate has been shown to enhance glycolytic flux in CD8+ T cells, supporting their activation, cytokine production and proliferation.^35^ This metabolic shift is crucial for their rapid expansion and sustained activity within the TME. SCFA influence glutaminolysis, a critical metabolic pathway that converts glutamine into intermediates fuelling the tricarboxylic acid (TCA) cycle, thereby supporting bioenergy, biosynthesis and redox homeostasis in proliferating cells, including activated CD8 T cells but also tumour cells (Figure 2).^36^

## 5 SCFA INCLUDING BUTYRATE, BOLSTER MEMORY T CELL FORMATION

Another critical factor influencing the efficacy of CIT is the formation of memory T cells. Evidence suggests that the microbiota can promote the differentiation and maintenance of these cells.^37^ Memory T cells proliferate rapidly proliferate upon re‐encounter with tumour‐associated neoantigens, mounting enhanced cytotoxic responses.^15^, ^37^ Among them, resident memory T cells (TRM), which persist in the tissues independently of the circulating T cell pool, provide an important localized and durable anti‐tumour defence.^38^ In 2022, Jaiswal et al. demonstrated that patients responding to immunotherapy exhibited an enhanced capacity to expand tissue‐resident and long‐term memory T cells.^39^ Using existing melanoma and cancer datasets, the study classified tumour infiltrating lymphocyte (TIL) responses in patients receiving CIT. A specific trajectory marked by high enrichment of memory and resident memory TILs was strongly associated with therapeutic response, highlighting the importance of these cell populations in mediating effective anti‐tumour immunity.^39^ Preclinical studies indicate that the differentiation of activated CD8+ T cells to memory cells is dependent on the presence of the gut microbiota and is further enhanced by butyrate.^40^ Bachem et al. (2019) showed that adoptive transfer of CD8+T cells into germ‐free recipients failed to generate memory cells, further supporting the essential role of the microbiota in memory formation. These findings suggest that the microbiota and its associated metabolites can support both short‐term and long‐term anti‐tumour immunity. Achieving optimal responses to CIT likely requires the establishment of a favourable immunogenic tumour microenvironment in conjunction with robust immunological memory. Microbiome‐derived metabolites are highly influential molecules that warrant further investigation to elucidate how they contribute to creating these conditions and enhancing the efficacy of current cancer therapies. Given the pleiotropic effects of SCFA however, their therapeutic development as potential CIT adjuvant must be precisely administered and contextually targeted. While SCFA can enhance the effector function, proliferation and memory formation of CD8+ T cells, indiscriminate or excessive supplementation may inadvertently provide metabolic substrate that support tumour cell growth and survival. Targeted delivery strategies that selectively deliver SCFA to immune cells, rather than tumour cells, represent a more effective and controlled approach.

## 6 CLINICAL AND PRECLINICAL EVIDENCE LINKING SCFA TO CIT OUTCOMES

Clinical studies examining the impact of SCFA on CIT responses remain limited.^20^, ^41^, ^42^ Botticelli et al. (2020) profiled faecal metabolites in 11 patients with non‐small cell lung cancer (NSCLC) undergoing CIT (2nd‐line nivolumab) and found that elevated levels of propionate, butyrate, lysine and nicotinic acid were associated with a progression‐free survival (PFS) exceeding 12 months.^20^ Nomura et al. assessed both faecal and plasma metabolites in a cohort of 52 patients (multiple tumour types), reporting that increased concentrations of faecal acetate, propionate, butyrate and plasma isovaleric acid correlated with longer PFS (Table 1).^42^ Conversely, Coutzac et al. (2020) demonstrated that high faecal and serum levels of butyrate and propionate were associated with therapeutic resistance to CTLA‐4 blockade and an increased proportion of Tregs in mice and humans.^41^ Supplementation of sodium butyrate in the drinking water diminished the efficacy of anti‐CTLA‐4 treatment in MC38 and MCA101_OVA_ mouse models.

**TABLE 1:** Metabolites are predictors of response in checkpoint inhibitor therapy.

	Disease Control	Disease Progression	Pathway	Tumour Type	Reference
**Measured in Serum**	Tryptophan, IDO	Increased Kyn/Trp ratio, Downstream tryptophan metabolites	Tryptophan catabolic pathway	Multiple	^20^, ^21^, ^22^, ^23^, ^46^
	Lactate/Pyruvate	Pyruvate	Glycolysis	NSCLC, Melanoma	^25^, ^65^
	Hypoxanthine Histidine		Adenosine metabolism	NSCLC	^20^
		Sphingolipid species, Myelin‐related metabolites	Sphingolipid metabolism	Melanoma	^25^, ^54^
		Alanine	Glycolysis		^65^
	Short chain fatty acids		Gut microbiome		^41^
	Branched chain amino acids		Support for mitochondrial function, support for protein formation		^22^, ^25^
**Measured in Stools**	SCFA including propionate, Butyrate, Lysine, nicotinic acid, Valeric acid, Acetic acid		Gut microbiome		^21^, ^41^
		2‐Pentanone (Ketone), Tridecane (Alkane), p‐Cresol	Gut microbiome		^2^

*Note*: Metabolites have been shown to be predictive of response to CIT. Key metabolites including Tryptophan, SCFA, branched chain amino acids as well as sphingolipid and myelin metabolites in melanoma could potentially be utilized as predictive biomarkers for patients undergoing CIT.

Although large‐scale targeted metabolomics studies are needed to clarify the relationship between SCFA and immunotherapy outcomes, SCFA deficiency and reduced microbial diversity have been consistently observed in autoimmune diseases, including multiple sclerosis (MS), type 1 diabetes, rheumatoid arthritis and psoriasis.^30^, ^43^, ^44^ In these contexts, SCFA promote regulatory lymphocyte function. For example, patients with MS exhibited significantly lower serum and faecal propionic acid compared to controls, and propionic acid supplementation increased Treg numbers.^44^ A small pilot study in patients with rheumatoid arthritis similarly reported a 45% increase in Tregs following propionic acid supplementation.^44^ These observations indicate that modulation of immune responses through SCFA supplementation can be effective in autoimmune diseases, although similar approaches have yet to be applied systematically in oncology.^30^, ^43^, ^44^

## 7 DIRECT SUPPLEMENTATION OF SCFA AS A POTENTIAL STRATEGY TO ENHANCE CIT

Direct supplementation of SCFA represents a promising avenue for enhancing CIT, although clinical studies in oncology are currently lacking. Several studies outside of the immunotherapy context have explored its immunomodulatory potential. A notable example is the Australian AusFAP study, designed to evaluate the effect of butyrate on polyp number in patients with familial adenomatous polyposis.^31^ In this double blind randomised, placebo‐controlled trial, patients are assigned to either 40 g/day of butyrylated high amylose maize starch (HAMSB) or low HAMSB, delivering butyrate to the large intestine, with the primary endpoint being the impact on large bowel polyp number.^31^

Further evidence of the immunological potential of SCFA supplementation comes from autoimmune disease studies. In MS, propionic acid supplementation has been shown to enhance the suppressive function of Tregs.^44^ Similarly, administration of high‐amylose maize‐resistant starch modified with acetate and butyrate in patients with type 1 diabetes increased SCFA concentrations in stools and plasma, promoting a more regulatory phenotype in both B and T cells.^43^ These findings underscore the need for systematic evaluation of SCFA administration in neoplasia, particularly in the context of CIT. Key questions for future research include determining which specific SCFAs to supplement, optimal dosages and duration, and the impact on the balance of cytotoxic CD8+ T cells versus Tregs. Understanding these parameters will be essential to harnessing SCFAs as modulators of anti‐tumour immunity.

Targeted delivery strategies that preferentially modulate immune cells within the TME, rather than systematically enhancing nutrient availability to malignant cells, represent a critical consideration for the translational application of SCFA‐based immunometabolic interventions. The role of nanomedicine has emerged as a transformative approach in CIT, particularly in modulating the TME to enhance immune responses while minimising off‐target side effects. Nanoparticles, such as polymeric micelles, nanogels, liposomes and magnetic structures, can be engineered to encapsulate SCFAs or other metabolic modulators.^45^ Surface functionalization allows selective targeting of specific immune cell populations within the TME, facilitating intracellular delivery.^46^ SCFA‐loaded nanoparticles can modulate the TME by promoting Treg differentiation, reduce their suppressive activity or enhance CD8+ cytotoxic T or natural killer (NK) cell function.^47^, ^48^, ^49^

## 8 TRYPTOPHAN CATABOLISM PREDICTS PRIMARY CHECKPOINT INHIBITOR THERAPY RESISTANCE

Tryptophan is an essential amino acid, whose catabolism regulates innate and adaptive immune responses.^50^, ^51^ In the context of CIT, increased kynurenine to tryptophan (Kyn/Trp) ratio reflects increased tryptophan catabolism and appears strongly correlated with poor prognosis^19^, ^20^, ^21^, ^52^ (Figure 1, Table 1). In humans, tryptophan comes from dietary intake and its free levels are determined by both oral intake and catabolism.^53^, ^54^ Tryptophan is degraded to kynurenine via the action of three rate limiting enzymes including indoleamine‐2,3 dioxygenase 1 and 2 (IDO1/2) in peripheral tissues and tryptophan‐2,3‐dioxygenase (TDO2) in the liver.^53^ Of these, the IDO‐1 pathway is the most fully characterized and described.^51^ Whilst only 5% of tryptophan is used for production of protein and neurotransmitters, 95% is then available for entry into the Trp and Kyn pathway.^55^ Cells expressing IDO therefore control tryptophan catabolism and play a key role in orchestrating immune responses, including the development of peripheral tolerance.^56^ The role of IDO in maintaining tolerance was demonstrated in pregnant mice exposed to IDO inhibitors, which resulted in rapid maternal T cell‐induced rejection and foetal loss.^57^ It has been recognised that IDO expression at the maternal–foetal interface prevents immunological rejection of the foetal allograft.^57^ Beyond this, we recognise the pivotal role that IDO plays in immunity due to its widespread expression in immune tissues including myeloid and stromal cells.^51^ Tryptophan catabolism and depletion is detrimental to T lymphocytes, which are exquisitely sensitive to tryptophan shortage.^51^, ^58^ For example, below a critical concentration, tryptophan deprivation causes a reproducible, cell cycle arrest for T cells. In this way, regulation of access to tryptophan can be a way through which IDO‐expressing antigen presenting cells can regulate T cell activation and response. The breakdown products of tryptophan include L‐Kyn, L‐hydroxykynurenine, 3‐hydroxyanthranilic acid, Quinolinic acid and Picolinic acid and these metabolites are known to have immunosuppressive properties, including induction of Treg differentiation, toxicity to lymphocytes and apoptosis of T effector cells.^58^ Apart from expression by immune cells, the majority of cancers also express IDO1 and this fact is exploited during the process of tumour immunoediting, during which enzyme expression is increased resulting in a rapid acceleration of immunosuppression and tumour growth Human metabolomics studies are concordant with these pre‐clinical findings and an elevated Kyn/Trp ratio or increased IDO activity have been associated with primary CIT resistance, poor prognosis and early progression of cancer.^20^, ^21^, ^52^ These findings highlight the pivotal role of the tryptophan pathway in regulating adaptive T cell responses, which are key drivers of CIT success.

## 9 INCREASED KYN/TRP RATIO AND IDO ACTIVITY MAY BE AN EARLY BIOMARKER OF CIT FAILURE

Increased Trp catabolism is associated with poor outcomes in patients undergoing CIT (Table 1).^19^, ^20^, ^21^, ^52^ In particular, elevated Kyn/Trp ratio or increased IDO activity are considered potential biomarkers of poor overall survival in solid tissue cancer patients receiving CIT, whereas increased tryptophan levels at baseline and lower levels of downstream tryptophan metabolites have been associated with disease control.^19^, ^20^, ^21^, ^52^Li et al. (2019) confirmed that Kyn/Trp ratio increases were an adaptive resistance mechanism associated with worse overall survival (OS) in both melanoma and renal cancer patients receiving CIT.^18^ Both melanoma and renal cell carcinoma patients who exhibited a Kyn/Trp ratio over 50% from baseline to week 4 had significantly worse overall survival compared to those with a decreased ratio.^18^ In melanoma, this translated to a median OS of 15.7 months versus > 38 months (*p* = 6 × 10^−5^), while in renal cell carcinoma the difference was 16.7 months versus 31.3 months (*p* = 4 × 10^−4^). More recently, Vilbert et al. (2023) examined 36 patients with cutaneous, uveal, or mucosal melanoma undergoing CIT and found that metabolites within the tryptophan‐kynurenine axis could differentiate cutaneous melanoma from the uveal and mucosal subtypes.^59^ This finding is clinically significant, as cutaneous melanoma is typically CIT responsive, whereas uveal and mucosal melanomas are largely CIT resistant and associated with poor prognosis.^59^

## 10 FAILURE OF IDO INHIBITORS TO DELIVER RESULTS IN CLINICAL PRACTICE

Despite the pivotal role of IDO1 in anti‐cancer immunity and the strong pre‐clinical rationale for IDO1 inhibition as an oncological target, clinical trials have not yielded the anticipated success.^27^, ^60^, ^61^ The phase III ECHO‐301/Keynote 252 trial of Epacadostat plus pembrolizumab versus pembrolizumab alone in patients with unresectable or metastatic melanoma showed no significant benefit with IDO1 inhibition.^62^ Similarly, multiple smaller randomised control trials investigating epacadostat or indoximod across various tumour types failed to improve objective response rates.^60^ These negative results have raised key questions regarding trial design, including the chosen dose (100 mg twice daily) was sufficient to suppress tryptophan catabolism, particularly given that checkpoint inhibitors can induce IDO activity. Moreover, intratumoural tryptophan levels and systemic tryptophan/kynurenine ratios were not assessed, and patient selection was not stratified by tumoural IDO1 expression. Addressing these limitations will be essential in future trials.

## 11 METABOLIC SIGNATURES ASSOCIATED WITH EITHER RESPONSE OR PROGRESSION TO CIT MAY BE USED TO STRATIFY PATIENTS AND PROVIDE INSIGHT INTO METABOLIC DEMANDS

Both CIT and chemotherapy reshape systemic metabolic profiles. CIT does so through immune‐mediated metabolic rewiring (tryptophan–kynurenine, glucose, glutamine), whereas chemotherapy exerts broader systemic effects through direct cytotoxicity and mitochondrial damage. These metabolic changes are increasingly studied as biomarkers of treatment response and targets for therapeutic modulation. Patients who respond to immunotherapy may ultimately have different immune adaptive capabilities to cope with the metabolic demands of the TME. The key characteristics of this may include the way in which T cells compete for and utilise glucose, amino acids and other key metabolites.^23^, ^63^ It is well known that the TME is a hostile landscape characterised by poor blood supply, hypoxia and lactic acidosis. For many years, research has focused on the metabolic aberrations that are unique to neoplasia, such as the Warburg effect, which reflects the fact that neoplastic tissue preferentially relies on glycolysis for energy.^63^ More recently, research efforts have increasingly turned to identifying how these metabolic changes affect immune cell function with the ultimate aim of restoring cytolytic activity of effector T cells, given that these cells have a natural capability to undergo metabolic rewiring or re‐programming.^64^, ^65^ Targeting the metabolic fitness of T cells is therefore a new and alternative way of enhancing the efficacy of immunotherapy.^65^, ^66^ Immune cells must compete within the hostile TME for vital nutrients and either adapt or risk dysfunction and exhaustion. Metabolomics can help confirm how localized nutrients are consumed in cancer and may be able to stratify patient cohorts into good/poor CIT responders in the pre‐treatment setting (Figure 1). The main groups of metabolites that have been studied include glucose, amino acids and lipids.^19^, ^67^, ^68^ The function of immune cells is impacted by this metabolic chaos of the TME and the field of immunometabolism is gaining momentum as an important research target.^64^, ^66^, ^69^ Glycolysis is one of the most studied metabolic pathways that is exploited by neoplasia.^63^ The Warburg effect has been well characterized as the process of preferentially utilising anaerobic glycolysis despite normally functioning mitochondria.^63^ This is believed to be advantageous in neoplasia, serving multiple functions including supporting the tremendous energy requirements of cancer, acidification of the environment and as a direct form of signal transduction.^63^ The end products of anaerobic glycolysis are pyruvate and lactate and their concentration can be assessed via metabolomics (Table 1). Triozzi et al. (2022) evaluated a group of melanoma patients (*n* = 40) undergoing CIT and evaluated both the bioenergetic signatures of immune cells (peripheral blood mononuclear cells PBMC) together with the plasma metabolomic profiles of responders compared to non‐responders. In this study, pyruvate was significantly lower in responders (*p* < 0.05) whereas lactate was significantly higher (*p* < 0.05).^23^ The responder population had an elevated ratio of lactate to pyruvate, which the authors favoured to reflect metabolic reprogramming of T cells towards a glycolytic pattern which would suggest improved priming to generate an effective effector T cell response. In a different study, Ghini et al. (2020) looked at a population of 53 patients with NSCLC.^70^ The serum samples of non‐responding patients were characterized by significantly higher levels of pyruvate, alanine as well as non‐significantly higher levels of lactate and glycine. The authors postulated that these findings reflected increased glycolysis within the hostile TME with an inability to overcome the metabolic challenges imposed by the tumour. It is important to reflect that the interpretation of metabolomics studies remains one of the most challenging aspects of this field and further studies are needed with blood and faecal collections at several different time points during treatment. Amino acid metabolism in the TME plays a role in tumour development and progression as branched chain amino acids are utilised by tumours to promote cell growth.^24^ Several metabolomics studies have confirmed significantly higher levels of branched chain amino acids (BCAA) for responders compared to non‐responders in populations of melanoma and NSCLC patients^19^, ^21^, ^52^, ^71^ (Figure 1). Branched chain amino acids are also important to the optimal metabolic functioning of immune cells including lymphocytes, which use BCAA for incorporation into protein, DNA and RNA in the setting of an evolving immune response.^19^ A recent study has utilized PP2Cm‐deficient mouse model has shown that impaired BCAA degradation led to enhanced anti‐tumour activity due to hyper‐activity of CD8+T cells.^72^, ^73^ In this mouse model, PP2Cm‐/‐mice are characterized by an impaired ability to breakdown BCAA and are therefore characterized by increased plasma and intracellular levels of BCAA which the authors postulated led to changes in TIL Cd8+T cell metabolic function. Furthermore, these effects synergized with CIT. Lipids including fatty acids, cholesterol, phospholipids and sphingolipids form a further area of interest in onco‐immunology.^74^ Lipids have vital roles in cellular function including maintenance of cell membrane structure and as an energy source in cells and lipid metabolism is known to be dysregulated in the setting of malignancy. Triozzi et al. (2022) reported elevated long chain fatty acids in melanoma responders.^23^ In contrast, non‐responders were characterized by significantly higher levels of sphingomyelin. Vilbert et al. (2023) similarly reported that uveal melanoma, which is known to be quite CIT resistant was similarly characterized by elevated sphingolipid species (*p *< 0.0001).^59^ Sphingolipid metabolism is known to be dysregulated in melanoma and is associated with disease progression.^75^ Mock et al. (2019) looked at patients with urological cancers receiving CIT and noted that serum very long chain fatty acid‐containing lipids could predict response to CIT in urologic cancers and the authors postulated that this may reflect a switch to fatty acid catabolism in T cells.^22^

A central question in immunometabolism pertains to the optimizing energy pathways for T cells in terms of their ability to continue to function as effector and cytotoxic cells. T‐cell metabolism is dynamic depending on the activation state of the cell with a quiescent T cell having lower energy demands compared to an activated cytotoxic CD8+ T cell. Triozzi et al. (2022) characterised bioenergetic, metabolic and genetic signatures associated with CIT response in melanoma patients.^23^ In this study, peripheral blood mononuclear cells of responding patients had higher reserve respiratory capacity and higher basal glycolytic activity which the authors felt reflected improved cellular bioenergetics. The authors postulated that responders may therefore be better positioned to execute glycolysis to generate an anti‐tumour response compared to non‐responders.

Serum metabolomics, typically performed via liquid chromatography‐mass spectrometry (LC‐MS), gas chromatography‐MS, or nuclear magnetic resonance spectroscopy, captures system‐wide metabolic alterations by detecting hundreds to thousands of metabolites, enabling broad biomarker discovery in clinical contexts such as immunotherapy response monitoring. However, while this approach offers high throughput and accessibility, it lacks the fine resolution necessary to resolve cell‐type or spatial metabolic heterogeneity within tissues or the tumour microenvironment.^76^, ^77^ Emerging modalities such as single‐cell and spatial metabolomics now allow unprecedented dissection of metabolic dynamics at the level of individual cells and specific tissue regions, exposing intratumoural and immune‐cell–specific metabolic variations that serum‐level analysis cannot discriminate.

## 12 FUTURE OPPORTUNITIES AND PERSPECTIVES

CIT has revolutionized cancer treatment and a plethora of other immune based therapies—CAR‐T cells to vaccines are currently being explored as potential therapeutic options for cancer patients. Whilst metabolic barriers are known to exist within the TME, for many years these have been identified and explored from the perspective of the tumour and only recently have these been reconsidered from the perspective of their impact on immunometabolism and the function of the immune system. Metabolomics is a relatively new field in biology that allows us to study the interplay of metabolites within a complex biological system and can help us to understand and contextualise the immunometabolic changes that are shaping treatment response. For example, increased Kyn/Trp ratio and IDO activity are poised to become early predictive biomarkers for CIT failure and disease progression. SCFA and the gut microbiome are clearly important in achieving functional immunity and this maybe through their effects on shaping balanced ratios of T effector cells/Tregs, enhancing cytotoxicity, alterations of the TME and enhancing memory function of T cells. Initial metabolomics data supports higher concentrations of SCFA as being positively correlated with increased PFS although further high‐volume studies are needed. Systemic metabolic readouts are increasingly recognised as predictors of immune‐related adverse events (irAEs) in patients receiving CIT. Elevated kyn/Trp ratios, reflecting enhanced IDO1 activity, have been linked to higher irAEs.^78^ Altered lipid metabolism, including phospholipid and fatty acid profiles can distinguish patients who develop colitis or hepatitis during PD‐1/PD‐L1 blockade.^79^ Moreover, baseline metabolomic signatures encompassing amino acid and bile acid derivatives have shown predictive value for irAEs in prospective studies.^80^ Immunometabolism has emerged as a promising therapeutic target in its own right. The capacity of T cells to undergo metabolic reprogramming highlights the potential to restore cellular fitness and thereby enhance the efficacy of CIT. Looking ahead, there are substantial opportunities to integrate metabolomic profiling into clinical practice, enabling patient responses to be evaluated not only by conventional measures of efficacy but also through the lens of metabolic adaptation and resilience.

## AUTHOR CONTRIBUTIONS

*Conceptualization*: Agnieszka Beata Malczewski, Natkunam Ketheesan, Severine Navarro and Jermaine Ig Coward. *Supervision*: Severine Navarro, Natkunam Ketheesan and Jermaine Ig Coward. *Writing—original draft*: Agnieszka Beata Malczewski, Severine Navarro, Natkunam Ketheesan and Jermaine Ig Coward. *Writing—review and editing*: Agnieszka Beata Malczewski, Severine Navarro, Natkunam Ketheesan and Jermaine Ig Cowa

## CONFLICT OF INTEREST STATEMENT

The authors declare no conflicts of interest.
